# Supplementary figures and images for: Activation of XBP1 but not ATF6α rescues heart failure induced by persistent ER stress in medaka fish
Source: Life Sci Alliance. 2023 May 9;6(7):e202201771. doi: 10.26508/lsa.202201771 (PMC10172766; doi:10.26508/lsa.202201771)

**Fig.1 Gel**

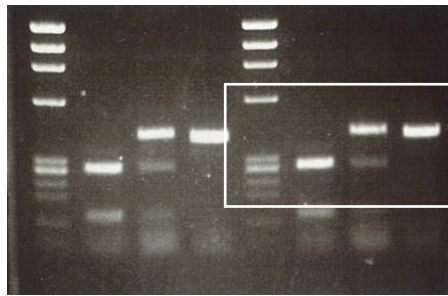

Supplement: Supplementary file 1 [file LSA-2022-01771_SdataF1.pdf]
