# Supplementary material for: Activation of XBP1 but not ATF6α rescues heart failure induced by persistent ER stress in medaka fish
Source: Life Sci Alliance. 2023 May 9;6(7):e202201771. doi: 10.26508/lsa.202201771 (PMC10172766; doi:10.26508/lsa.202201771)
Supplement: Supplementary file 12 [file LSA-2022-01771_TableS1.docx]

**Supplementary File 1 Names and sequences of various primers**

| Reagent type or resource | Designation | Source or reference | Identifier | Additional information |
| --- | --- | --- | --- | --- |
| Sequence- based reagent | β-actin cDNA Fw | Ishikawa et al., 2017 | qRT-PCR primer | CGGTATCCATGAGACCACCT |
| Sequence- based reagent | β-actin cDNA Rv | Ishikawa et al., 2017 | qRT-PCR primer | AGCACAGTGTTGGCGTACAG |
| Sequence- based reagent | GRP58 cDNA Fw | This paper | qRT-PCR primer | GCCCAAATACAACGAGCTGG |
| Sequence- based reagent | GRP58 cDNA Rv | This paper | qRT-PCR primer | TGGCTGTAGCGTCCATCTTG |
| Sequence- based reagent | BiP/GRP78 cDNA Fw | This paper | qRT-PCR primer | AGACAGAAGATTTCCAGGCCA |
| Sequence- based reagent | BiP/GRP78 cDNA Rv | This paper | qRT-PCR primer | CGCCTGCACTTCCATAAAGC |
| Sequence- based reagent | GRP94 cDNA Fw | This paper | qRT-PCR primer | AGACAGGATAGAGCGCATGC |
| Sequence- based reagent | GRP94 cDNA Rv | This paper | qRT-PCR primer | TCTGGTTCCTCCTCTGGTTC |
| Sequence- based reagent | ORP150 cDNA Fw | This paper | qRT-PCR primer | ACATCCAGGATCTGACAGCC |
| Sequence- based reagent | ORP150 cDNA Rv | This paper | qRT-PCR primer | AGGTTGTGTCTTTTCCGTCG |
| Sequence- based reagen | ERp72 cDNA Fw | This paper | qRT-PCR primer | CGACTTCGACTCGGAGGTTC |
| Sequence- based reagen | ERp72 cDNA Rv | This paper | qRT-PCR primer | GGCTGGGACTTGATGATGGG |
| Sequence- based reagent | CRT1  cDNA Fw | This paper | qRT-PCR primer | AGGAAGTGGGCAATGACACC |
| Sequence- based reagent | CRT1  cDNA Rv | This paper | qRT-PCR primer | CCTCTCCTCTTCGTCTTGGC |
| Sequence- based reagent | CRT3-1  cDNA Fw | This paper | qRT-PCR primer | CGGAAGACGTGGGCAAAGAG |
| Sequence- based reagent | CRT3-1  cDNA Rv | This paper | qRT-PCR primer | CGCTTCAACTCGTCCTGCTC |
| Sequence- based reagent | CRT3-2  cDNA Fw | This paper | qRT-PCR primer | CAAAGAGGCTGAGGAGGTCG |
| Sequence- based reagent | CRT3-2  cDNA Rv | This paper | qRT-PCR primer | TGTCCTCTTGGTCATCCCTC |
| Sequence- based reagent | HRD1  cDNA Fw | This paper | qRT-PCR primer | CCCAGCAGCTCTGAGAAGA |
| Sequence- based reagent | HRD1  cDNA Rv | This paper | qRT-PCR primer | GAGTCAGCGGGTAGAGAGGA |
| Sequence- based reagent | SEL1L  cDNA Fw | This paper | qRT-PCR primer | CCTTGTGTACACCCTGCAGTA |
| Sequence- based reagent | SEL1L  cDNA Rv | This paper | qRT-PCR primer | AGAAGCTGGTCCAGGTCAAC |
| Sequence- based reagent | Derlin1  cDNA Fw | This paper | qRT-PCR primer | AACTGACGGGAAACCTGGTG |
| Sequence- based reagent | Derlin1  cDNA Rv | This paper | qRT-PCR primer | TGTTGGGGAAGAACCGATAC |
| Sequence- based reagent | EDEM1  cDNA Fw | This paper | qRT-PCR primer | AAGAAGATCGCATGGAGAGC |
| Sequence- based reagent | EDEM1  cDNA Rv | This paper | qRT-PCR primer | TGTGACCCTCAGTGGTAAAG |
| Sequence- based reagent | XTP3B  cDNA Fw | This paper | qRT-PCR primer | CTGCTACGGAAAGCATGTCC |
| Sequence- based reagent | XTP3B  cDNA Rv | This paper | qRT-PCR primer | CAACGTTCTTCTTGGCCCAG |
| Sequence- based reagent | OS9  cDNA Fw | This paper | qRT-PCR primer | CAGGGAGCTGCTCATTAACC |
| Sequence- based reagent | OS9  cDNA Rv | This paper | qRT-PCR primer | GATTCCTCCCGATTCTCTCC |
| Sequence- based reagent | Gp78  cDNA Fw | This paper | qRT-PCR primer | GCTGAGGAAAGAGGAGATGC |
| Sequence- based reagent | Gp78  cDNA Rv | This paper | qRT-PCR primer | TCCTCGTCCTGATTCTCTGG |
| Sequence- based reagent | DESMA Fw | This paper | Genomic  PCR primer | TTTGAATTCCCTGCAAACAACCAGCCAAC |
| Sequence- based reagent | DESMA Rv | This paper | Genomic  PCR primer | AAATCTAGAGCTTCTTTACATGATGTCTTGCTG |
| Sequence- based reagent | MTHFD2 Fw | This paper | Genomic  PCR primer | TTTGAATTCTGGCTGATATTGTCACTTTCCC |
| Sequence- based reagent | MTHFD2 Rv | This paper | Genomic  PCR primer | AAATCTAGATGTCACTGTAGCTCAAGCAC |
| Sequence- based reagent | SOCS3 Fw | This paper | Genomic  PCR primer | TTTGAATTCAAAGTGGGGACAGCGCCATG |
| Sequence- based reagent | SOCS3 Rv | This paper | Genomic  PCR primer | AAATCTAGACCCTTCATGGCGTCAGTCCAG |
| Sequence- based reagent | CYP24A1 Fw | This paper | Genomic  PCR primer | TTTGAATTCATGAGGGCGCAGGTCCAAAAAG |
| Sequence- based reagent | CYP24A1 Rv | This paper | Genomic  PCR primer | AAATCTAGAAGCTGTCTGTCGTCACATCCAG |
| Sequence- based reagent | SGK1 Fw | This paper | Genomic  PCR primer | TTTGGATCCATGGCCGTGACTGAGGCTGG |
| Sequence- based reagent | SGK1 Rv | This paper | Genomic  PCR primer | AAATCTAGAAATCCAGGGGTCGGAGGTGC |
| Sequence- based reagent | CTHL Fw | This paper | Genomic  PCR primer | TTTGAATTCTTAGGAAACACGCCGCGATG |
| Sequence- based reagent | CTHL Rv | This paper | Genomic  PCR primer | AAACTCGAGCAATCCACTGAGGGGACCTC |
| Sequence- based reagent | PHGDH Fw | This paper | Genomic  PCR primer | TTTGAATTCCCACGCATGCTTTCAGCTTG |
| Sequence- based reagent | PHGDH Rv | This paper | Genomic  PCR primer | AAATCTAGATCCGGAGTGGTTAAGTGCTG |
| Sequence- based reagent | ADAMTS16 Fw | This paper | Genomic  PCR primer | TTTGAATTCCGTCAGACATGAGCGGAACC |
| Sequence- based reagent | ADAMTS16 Rv | This paper | Genomic  PCR primer | AAATCTAGACGGACTGGTTCAGTCTGCAAC |
| Sequence- based reagent | NPPC Fw | This paper | Genomic  PCR primer | TTTGAATTCAGCGCCAAGAGGAATCCCAG |
| Sequence- based reagent | NPPC Rv | This paper | Genomic  PCR primer | AAATCTAGATTCACAGCGTTGCTCCAACC |
